# ChatGPT versus UpToDate in Preclinical Medical Education: Cross-Sectional Analysis Using Term Frequency–Inverse Document Frequency Cosine Similarity

**DOI:** 10.2196/82885

**Published:** 2026-03-20

**Authors:** Shankar S Thiru, Nicholas E Aksu, Matthew Chiang, Daniel O Gallagher, Mary Furlong, Elizabeth R Prevou, Akhil Jay Khanna

**Affiliations:** 1 Georgetown University School of Medicine, 3800 Reservoir Rd, Washington, DC, 20007, United States; 2 Department of Orthopaedic Surgery, MedStar Georgetown University Hospital, Washington, DC, United States; 3 DataChat Inc., Madison, WI, United States; 4 Rothman Orthopaedic Institute, Thomas Jefferson University Hospital, Philadelphia, PA, United States; 5 Department of Pathology and Laboratory Medicine, Georgetown University School of Medicine, Washington, DC, United States; 6 The George Washington University School of Medicine and Health Sciences, Washington, DC, United States

**Keywords:** artificial intelligence, medical education, preclinical, ChatGPT, UpToDate

## Abstract

**Background:**

Generative artificial intelligence tools such as ChatGPT are increasingly used by medical students for self-directed learning. Although these models demonstrate linguistic fluency, their reliability as supplementary resources for preclinical education remains uncertain. In particular, comparisons with evidence-based references such as UpToDate are lacking.

**Objective:**

This study evaluated the similarity between responses generated by ChatGPT (with GPT-4o mini) and those from UpToDate to preclinical medical education questions to assess ChatGPT’s potential as an adjunctive learning tool.

**Methods:**

We conducted a cross-sectional comparison study using 150 first-order questions derived from a preclinical question bank at a single allopathic institution under the oversight of a medical educator with more than 25 years of teaching experience. Each question was entered into ChatGPT 10 times in separate chat sessions, and responses from UpToDate were retrieved from the most relevant articles. The responses were preprocessed through lemmatization, stop-word removal, punctuation removal, and numeric normalization. Similarity between ChatGPT and UpToDate responses was quantified using term frequency–inverse document frequency (TF-IDF) cosine similarity. To determine whether the observed similarities exceeded chance, ChatGPT outputs were compared with a null distribution generated from randomized text.

**Results:**

ChatGPT responses demonstrated statistically significant similarity to UpToDate in 59.3% (89/150) of questions. Across subject areas, pharmacology showed the highest concordance (mean cosine similarity 0.338, SD 0.134), followed by pathology (mean 0.321, SD 0.142), biochemistry (mean 0.296, SD 0.120), microbiology (mean 0.297, SD 0.108), and immunology (mean 0.275, SD 0.102). All subject-level similarity scores exceeded those generated from randomized text, confirming that the observed overlap was nonrandom.

**Conclusions:**

ChatGPT with GPT-4o mini exhibited moderate but meaningful alignment with UpToDate across preclinical topics, performing best in fact-based disciplines such as pharmacology. Although it is not a substitute for evidence-based resources, ChatGPT may serve as an accessible adjunctive tool for medical students. Integration into preclinical learning should be coupled with artificial intelligence literacy training to promote responsible use and critical appraisal.

## Introduction

Artificial intelligence (AI) refers to the ability of digital systems to perform tasks traditionally requiring human intelligence, such as pattern recognition, problem-solving, and decision-making [1-3]. One rapidly advancing branch of AI is generative AI, which includes large language models (LLMs) capable of producing novel text, images, or other media based on extensive training datasets. ChatGPT, an LLM developed by OpenAI, enables iterative question-and-answer dialogue with users and has generated significant interest for its potential applications in health care and education [4,5].

In medical education, ChatGPT has been investigated for several instructional benefits, including its ability to generate customized clinical cases, provide immediate feedback, and distill complex topics into accessible explanations [6]. Early studies have even demonstrated that ChatGPT can perform at or above passing thresholds for step 1 of the United States Medical Licensing Examination, suggesting proficiency with biomedical content integration [7]. At the same time, learners are increasingly incorporating generative AI tools into their study practices for tasks such as summarizing notes, preparing for board examinations, and conducting self-assessments [8-13]. This trend has raised important questions about the reliability, accuracy, and alignment of generative AI tools with evidence-based resources. Prior work indicates that although ChatGPT demonstrates linguistic fluency and strong knowledge recall, it can underperform in tasks requiring algorithmic reasoning, symbolic processing, or deep, domain-specific expertise [14].

Despite growing attention to the use of generative AI in clinical training and high-stakes assessment contexts, the role of ChatGPT for students engaged in the preclinical curriculum remains largely unexplored. Equally important, there have been no studies directly comparing ChatGPT to UpToDate, a widely adopted, evidence-based medical knowledge database that serves as a cornerstone resource for medical trainees. Understanding how ChatGPT’s responses align with an established standard is essential to determining whether it may serve as a credible supplementary tool in early medical education.

Accordingly, this study evaluated ChatGPT (with GPT-4o mini) in comparison with UpToDate across a series of preclinical curricular questions by examining the degree of similarity between ChatGPT-generated responses and content provided by UpToDate to determine whether generative AI can serve as a reliable adjunct to evidence-based resources for first- and second-year medical students.

## Methods

### Study Design

**Figure 1.**
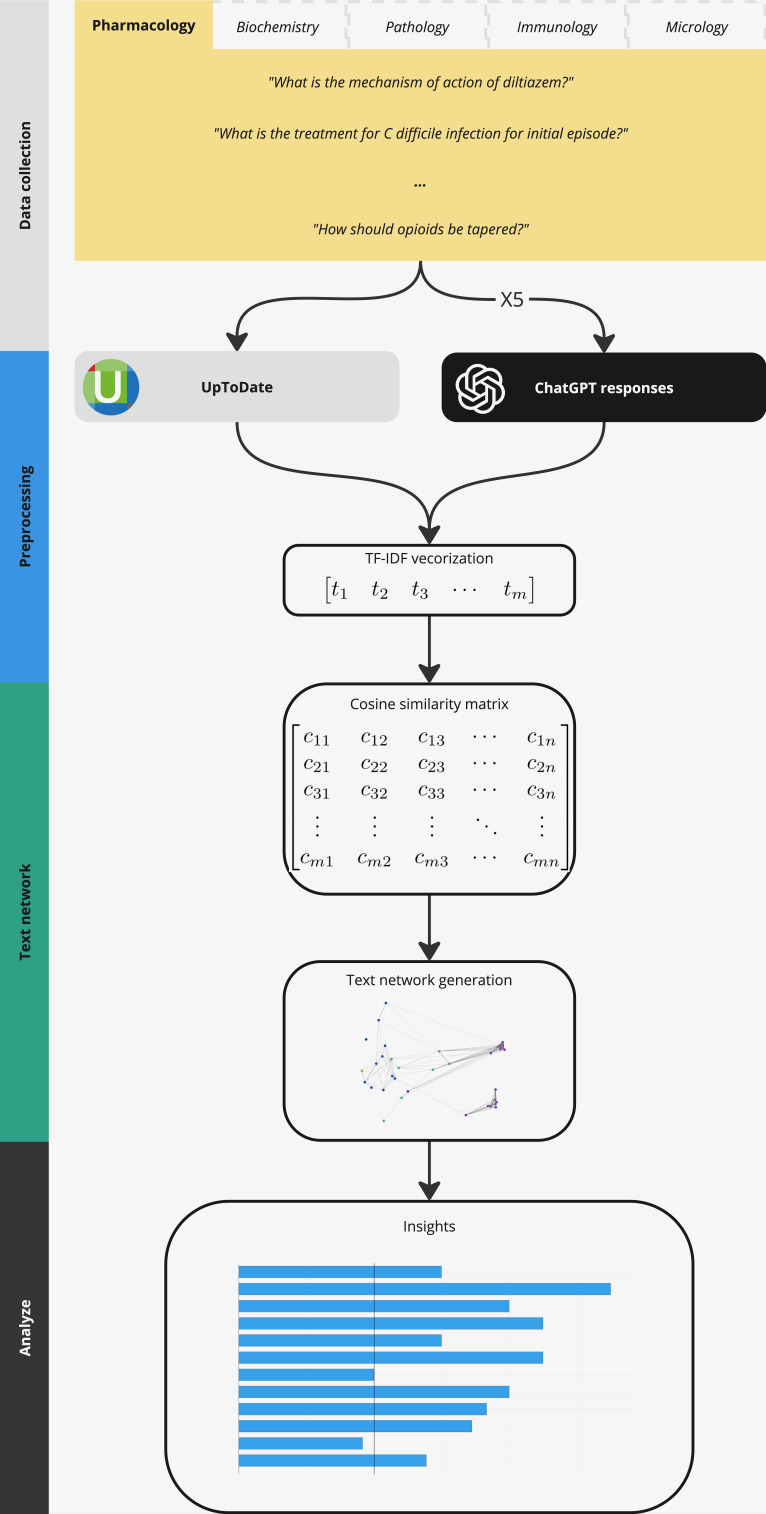
The term frequency–inverse document frequency (TF-IDF) cosine similarity and text networks for preclinical subject questions and aggregate ChatGPT responses in biochemistry. The statistical baseline was determined from textual similarity to randomly generated text.

This was a cross-sectional comparison study (Figure 1). We examined how ChatGPT’s free version (GPT-4o mini) responded to preclinical medical science questions compared with the medical knowledge database, UpToDate. UpToDate was selected because it is a continuously updated, physician-authored, and peer-reviewed medical knowledge resource that compiles primary literature into standardized, consensus-based explanations. Although primarily designed for clinical decision support, UpToDate extensively covers foundational biomedical concepts that directly overlap with preclinical curricula, including pathophysiology, pharmacologic mechanisms, microbiology, and immunology. Prior studies have used UpToDate as a reference standard when evaluating the accuracy of digital health tools and clinical decision-support systems, supporting its use as a high-quality comparator for authoritative medical knowledge [15-17].

### Question Selection

A total of 150 questions were derived from a preclinical medical education question bank used at Georgetown University. Questions were selected to reflect core content areas common to standard US preclinical curricula, including biochemistry, immunology, microbiology, pharmacology, and pathology. To approximate real-world information-seeking behavior, this study specifically included first-order questions (ie, those querying a single fact or concept) comparable in scope and difficulty to material found in step 1 of the foundational United States Medical Licensing Examination, which reflects typical student search patterns. Although the question set was not formally validated across multiple institutions, its content and difficulty were reviewed to ensure alignment with nationally standardized learning objectives. Question selection and categorization were conducted under the oversight of a board-certified medical educator with over 25 years of experience in undergraduate medical education, ensuring curricular relevance and pedagogical appropriateness for the preclinical learner.

### Data Collection

Each question was entered verbatim into ChatGPT 10 separate times, with each entry conducted in a new chat window and without a logged-in user to prevent prior prompts from influencing responses. Multiple samples were collected to account for the inherent stochasticity of ChatGPT, as repeated queries can yield variable phrasing or content for identical prompts. GPT-4o mini was selected because it is the free, publicly available version and therefore the version most likely to be used by students during self-study.

For UpToDate, each question was entered into the search bar function (using Google Chrome version 131.0, in incognito mode, with the browsing history cleared). To standardize data collection, only the overview or explanatory sections of the most relevant UpToDate articles were extracted for analysis. When multiple UpToDate articles were relevant to a given question, the section deemed most directly responsive to the query was selected based on content specificity and scope. Selection criteria for articles included (1) direct factual or mechanistic information, (2) conciseness and clarity of explanation, and (3) being authored or reviewed by recognized experts in the field, as indicated by UpToDate’s editorial process. For example, if a pharmacology question asked about the mechanism of action of a specific drug class, the section explicitly describing the drug’s pharmacodynamics was selected over general overviews or therapeutic context.

UpToDate responses were collected once, reflecting its status as a static, curated knowledge source. Sensitivity analyses using subsets of ChatGPT outputs (eg, first, median, and maximum similarity scores across the 10 samples) confirmed that overall trends and domain-level patterns remained consistent, ensuring comparisons captured meaningful alignment rather than artifacts of sampling variability (Table 1). This approach ensured that responses represented the type of summary text a student would likely consult.

**Table 1.** Sensitivity analysis of ChatGPT term frequency–inverse document frequency similarity scores.

Domain	First	Median (SD)	Maximum to minimum
Pharmacology	0.330	0.338 (0.014)	0.355‐0.310
Pathology	0.312	0.321 (0.015)	0.340‐0.290
Biochemistry	0.290	0.296 (0.013)	0.310‐0.265
Microbiology	0.290	0.297 (0.016)	0.310‐0.275
Immunology	0.270	0.275 (0.011)	0.290‐0.250

### Text Preprocessing (Normalization)

Responses were normalized to allow meaningful comparison because natural language allows for variation (eg, “run,” “ran,” “running” all convey the same core concept). Normalization included lemmatization, which reduced words to a common base form (eg, “runs” to “run”*)*; stop-word removal, which eliminated common filler words such as “the” or “and” that do not affect meaning; syntax removal, which removed punctuation and formatting; and number handling, in which all numeric values were dropped to prevent disproportionate influence from isolated numbers (eg, drug dosages). These steps reduced irrelevant variation while preserving core content.

### Text-to-Network Conversion and Similarity Analysis

Each processed response was converted into a vector using term frequency–inverse document frequency (TF-IDF). TF-IDF weights each word according to both its frequency within a response and its rarity across the dataset, ensuring that uncommon but meaningful words carry greater weight while common words contribute less.


$$ChatGPTnode:v_{i}^{*}$$

$$UpToDatenode:v_{j}$$

$$Element-wise\;mean\;of\;weights:\;w_{j}=\;\frac{1}{N*M}\sum_{i=1}^{N}\sum_{j=1}^{M}e_{ij}$$

To compare two responses (eg, ChatGPT vs UpToDate), we calculated cosine similarity between their TF-IDF vectors. Cosine similarity ranges from 0 (no similarity in text) to 1 (identical text). For example, high similarity (≈0.85) is observed between “doctors treat patients in hospitals” versus “physicians treat patients in outpatient hospitals,” whereas low similarity (≈0.30) is observed between “doctors treat patients in hospitals” and “engineers design bridges with software.*”* Thus, higher scores indicate greater overlap in informational content.

To account for variability in ChatGPT outputs, 5 responses per question were compared to the corresponding UpToDate text, and the results were aggregated into an average similarity score per question. Aggregated scores were then averaged within each subject area to examine differences across disciplines, consequently using the same textual information but different corpora composition. Definitions of terminology are found in Table 2.

**Table 2.** Definitions of terms for analytical tools used in the analysis.

Term	Definition
TF-IDF^a^	A statistical, quantitative measure used to evaluate the importance of a word in a document relative to a collection of documents (corpus). It combines two components: the frequency of a term in a document (term frequency) and the inverse frequency of that term across all documents in the corpus (inverse document frequency). High TF-IDF values indicate words that are unique to specific documents.
Text vector network	A network representation of text where each node is a vector representation of words, phrases, or entire documents. These vectors are typically derived using the TF-IDF values of a text, and the edges represent relationships such as the similarity between the vectors. This type of analysis helps capture semantic and contextual relationships within large text corpora.
Cosine similarity matrix	A matrix used to measure the cosine of the angle between vectors representing text in a vector space model. The cosine similarity ranges from −1 (completely dissimilar) to 1 (identical). In text analysis, it is commonly used to assess the similarity between documents or terms based on their vectorized representations. A higher value corresponds to a higher similarity between two texts.
Text network analysis	An overall approach of analyzing the relationships between words, phrases, or documents using text vector networks and cosine similarity matrices. This approach helps uncover patterns, structures, and clusters in textual data.

a TF-IDF: term frequency–inverse document frequency.

### Statistical Analysis

To determine whether observed similarities exceeded chance, we created a null distribution by generating 1000 randomized text samples that matched the length and word distribution of UpToDate outputs (Multimedia Appendix 1). The TF-IDF cosine similarity scores were calculated for these random texts against UpToDate. This approach enabled comparison of ChatGPT and UpToDate similarity with a baseline random similarity distribution. Statistical significance was defined as a probability value of less than .05, with ChatGPT and UpToDate similarity exceeding the 95th percentile of random-text similarities.

### Study Outcomes

The primary outcome was the similarity (ie, cosine similarity score) between ChatGPT and UpToDate responses at the individual question level. The secondary outcome was the difference in similarity across preclinical subject categories.

### Ethical Considerations

This study did not involve human or animal subjects; therefore, ethical approval was not required.

## Results

**Figure 2.**
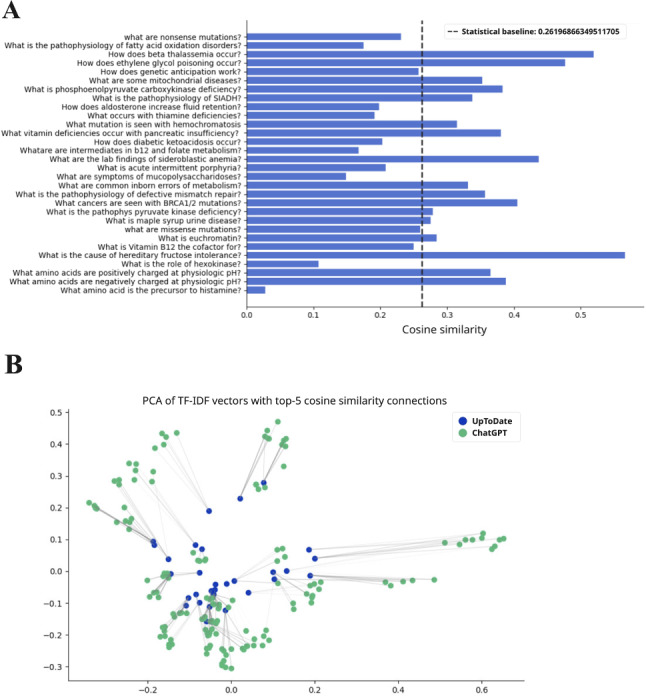
The TF-IDF cosine similarity and text networks for preclinical subject questions and aggregate ChatGPT responses for immunology. The statistical baseline was determined from textual similarity to a randomly generated text. PCA: principal component analysis; SIADH: syndrome of inappropriate antidiuretic hormone secretion; TF-IDF: term frequency–inverse document frequency.

**Figure 3.**
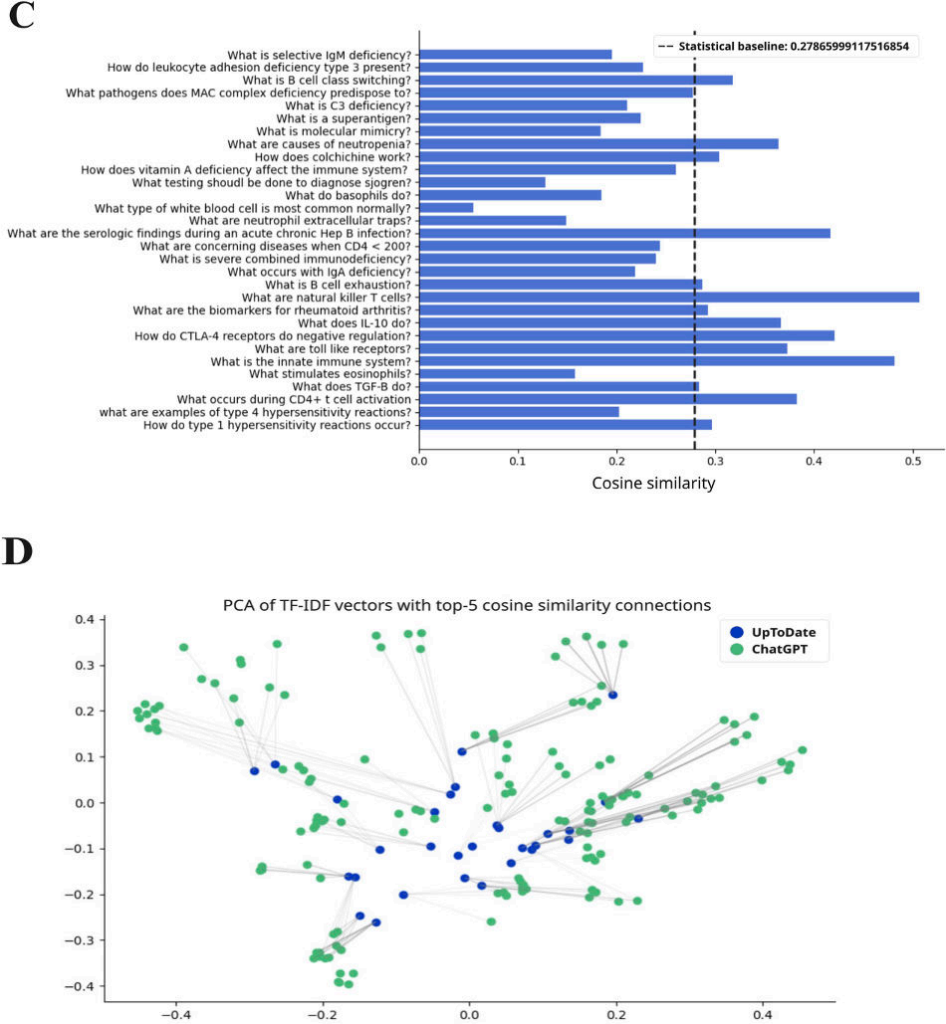
The TF-IDF cosine similarity and text networks for preclinical subject questions and aggregate ChatGPT responses for microbiology. The statistical baseline was determined from textual similarity to randomly generated text. lgM: immunoglobulin; MAC: membrane attack complex; PCA: principal component analysis; TF-IDF: term frequency–inverse document frequency.

**Figure 4.**
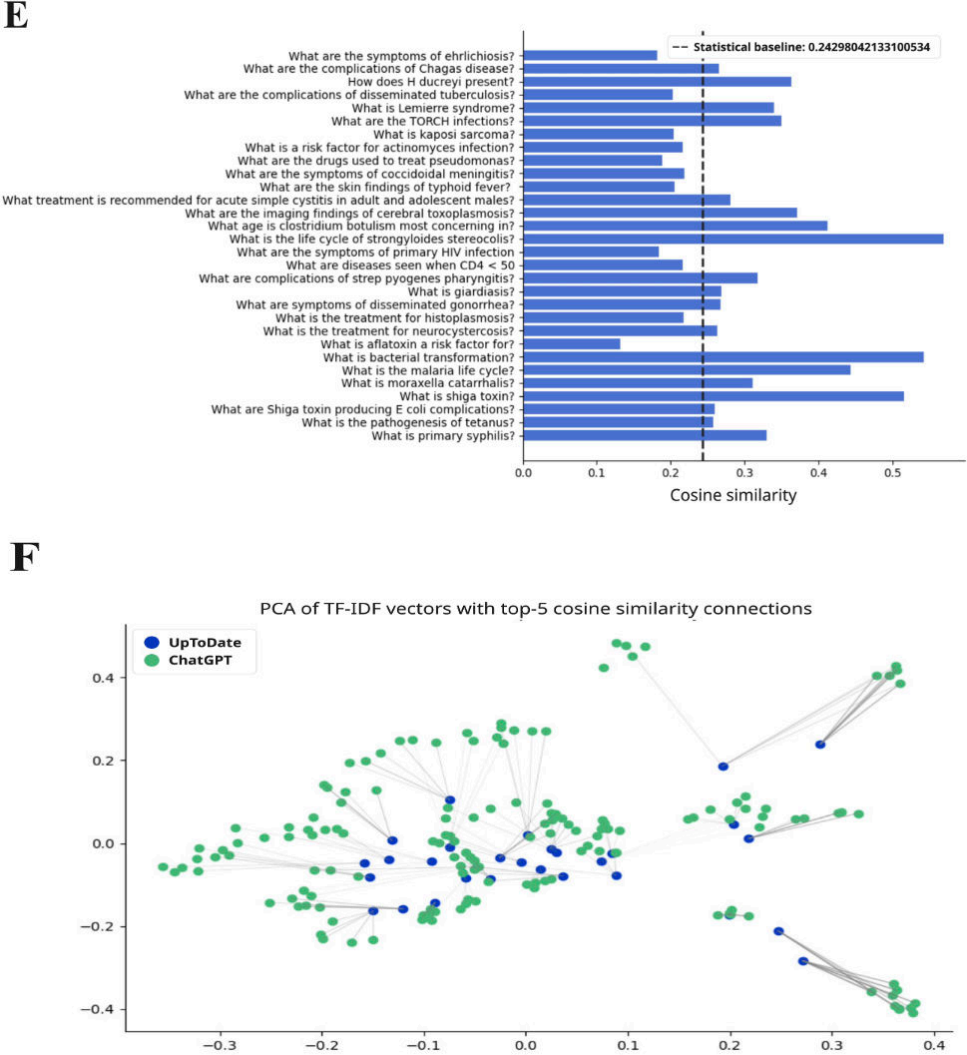
The TF-IDF cosine similarity and text networks for preclinical subject questions and aggregate ChatGPT responses for pathology. The statistical baseline was determined from textual similarity to randomly generated text. PCA: principal component analysis; TF-IDF: term frequency–inverse document frequency; TORCH: toxoplasmosis, other infections, rubella, cytomegalovirus, and herpes simplex virus.

**Figure 5.**
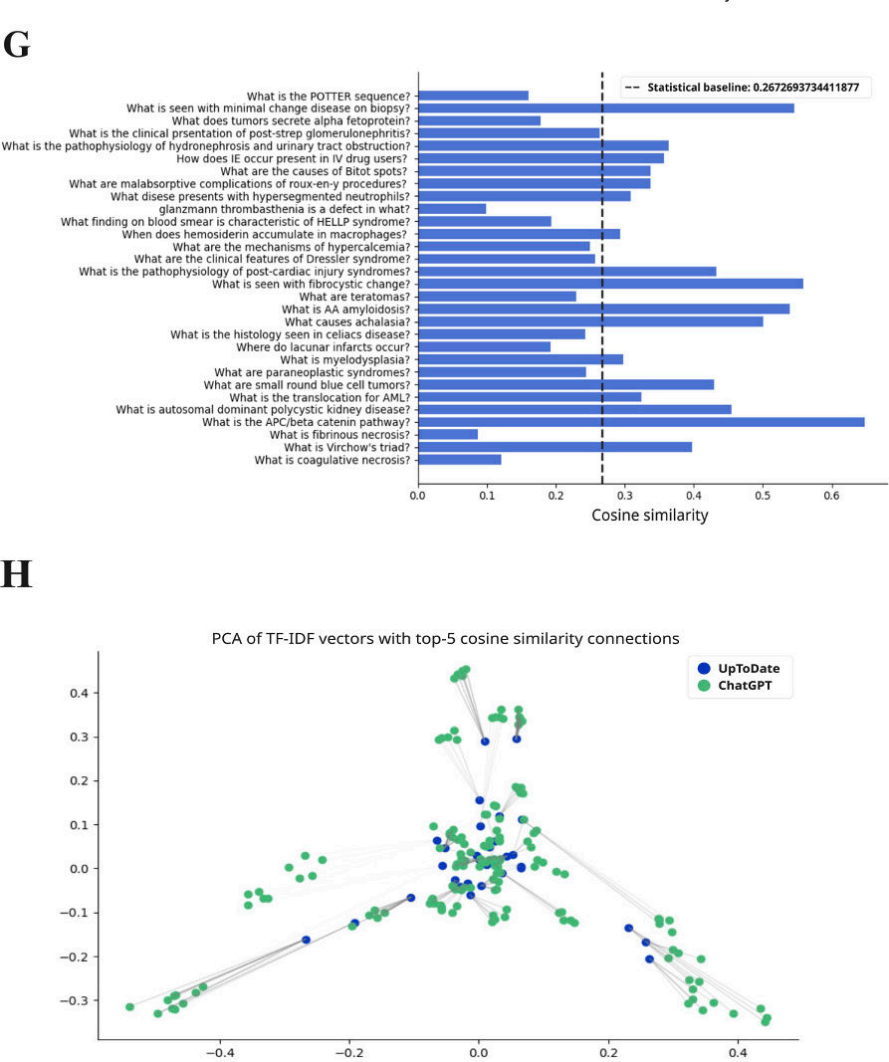
The TF-IDF cosine similarity and text networks for preclinical subject questions and aggregate ChatGPT responses for pharmacology. The statistical baseline was determined from textual similarity to randomly generated text. PCA: principal component analysis; TF-IDF: term frequency–inverse document frequency; IV: intravenous.

**Figure 6.**
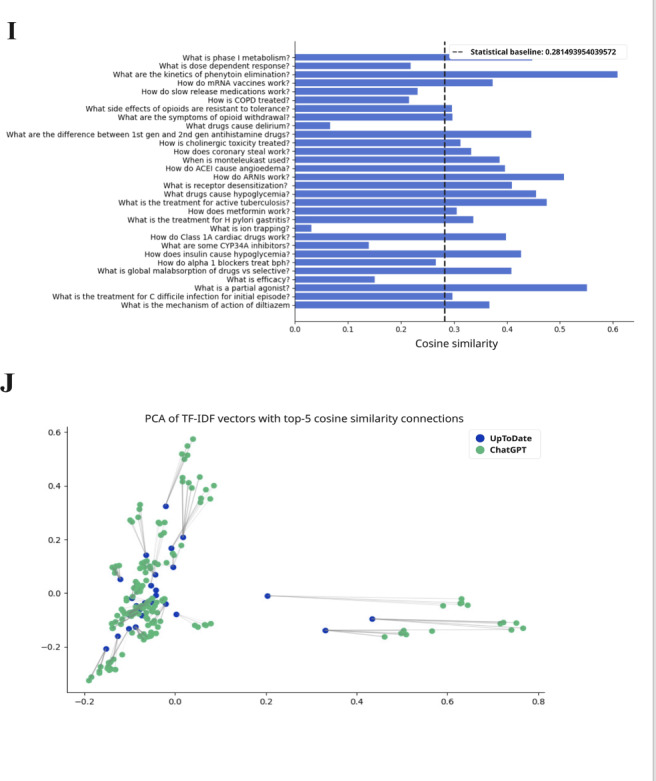
The overall cosine TF-IDF similarity between ChatGPT and UpToDate for the preclinical subjects of biochemistry, immunology, microbiology, pathology, and pharmacology is shown. Subjects are displayed in order from most to least similar. The statistical baseline was determined from textual similarity to randomly generated text. PCA: principal component analysis; TF-IDF: term frequency–inverse document frequency.

ChatGPT responses demonstrated moderate similarity to UpToDate across preclinical topics. Using TF-IDF cosine similarity, 89 of 150 questions (59.3%) produced ChatGPT responses that were statistically similar to those from UpToDate (Figures 2-6; precise values are available in Multimedia Appendix 2).

Performance was consistent across disciplines (Table 3). For example, 56.6% (17/30) of biochemistry questions and comparable proportions in immunology, microbiology, pharmacology, and pathology yielded statistically similar responses (*P*<.001).

**Table 3.** Frequency of questions (N=150) with similarity and nonsimilarity between ChatGPT and UpToDate for each preclinical subject.

Subject	Number of questions with similarity between ChatGPT and UpToDate, n (%)	Number of questions with no similarity between ChatGPT and UpToDate; n (%)
Biochemistry	17 (56.6)	13 (43.4)
Immunology	14 (46.7)	16 (53.3)
Microbiology	19 (63.3)	11 (36.7)
Pathology	17 (56.6)	11 (43.4)
Pharmacology	22 (73.3)	8 (26.7)

When mean similarity scores were analyzed, pharmacology achieved the highest concordance with UpToDate (TF-IDF mean 0.338, SD 0.134; *P*<.001), followed by pathology (mean 0.321, SD 0.142; *P*<.001), biochemistry (mean 0.296, SD 0.120; *P*<.001), microbiology (mean 0.297, SD 0.108; *P*<.001), and immunology (mean 0.275, SD 0.102; *P*<.001). These values consistently exceeded the similarity scores generated from randomized text baselines (range 0.243‐0.281 across subjects; 0.148 for domain-level aggregation), confirming that ChatGPT’s alignment with UpToDate reflected meaningful overlap beyond chance.

## Discussion

### Interpretation and Educational Implications

In the cross-sectional analysis, we found that ChatGPT demonstrated moderate but incomplete alignment with UpToDate across preclinical medical topics. Using TF-IDF cosine similarity, approximately 59% of ChatGPT responses were statistically similar to those generated by UpToDate, with consistent performance across disciplines. The highest concordance was observed in pharmacology. Importantly, similarity scores consistently exceeded randomized text baselines, indicating meaningful overlap beyond chance. However, more than one-third of responses diverged, underscoring important limitations in ChatGPT’s reliability as a standalone educational resource.

With medical students increasingly integrating generative AI into their learning routines, understanding both the strengths and shortcomings of LLMs has become essential. Concerns persist regarding the accuracy and transparency of ChatGPT, particularly given documented instances of fabricated references and confidently stated inaccuracies [18]. By directly comparing ChatGPT with UpToDate, a widely regarded gold standard resource in medical education, this study provides novel insights into the credibility and potential educational role of LLMs in the preclinical curriculum.

This study’s primary finding was that ChatGPT’s responses were statistically similar to those of UpToDate in nearly 60% of cases. This suggests that ChatGPT is capable of producing outputs that meaningfully overlap with an established, peer-reviewed knowledge source. Importantly, however, more than one-third of responses diverged, underscoring that ChatGPT cannot yet be viewed as a direct replacement for authoritative resources. Instead, its greatest value may lie in functioning as a supplementary study aid, particularly when learners seek rapid explanations, iterative clarification, or simplified summaries. Unlike UpToDate’s search-based retrieval of curated content, ChatGPT generates novel text in response to queries. This generative capacity could represent a shift in how students engage with knowledge; however, concerns about verifiability and the lack of time-stamped references remain major limitations to its educational utility.

ChatGPT demonstrated consistency across subject domains, with pharmacology achieving the highest similarity scores. This may reflect pharmacology’s reliance on factual, definitional content (eg, mechanisms of action, drug classes), which generative AI models are better able to reproduce. In contrast, immunology and microbiology demonstrated greater divergence, likely due to their reliance on nuanced processes and context-dependent reasoning. These differences emphasize that AI may be better suited for fact-based knowledge than for subjects requiring higher-order integration or precise mechanistic detail. Incorporating domain experts in the design and refinement of AI models, similar to the physician involvement that underpins UpToDate, may help address these gaps and improve domain-specific accuracy [19].

From an educational perspective, these findings suggest that ChatGPT could serve as a readily accessible, low-barrier tool for students seeking supplemental explanations or conceptual reinforcement. However, educators must guide learners in understanding its limitations. Training students in AI literacy—the ability to critically appraise, cross-reference, and contextualize AI outputs—will be crucial to prevent overreliance on potentially inaccurate content. Future curricular integration of AI tools should emphasize transparency, verification against primary or trusted secondary sources, and responsible use in complementing (not replacing) established references.

### Limitations

This study has several limitations. First, TF-IDF cosine similarity quantifies overlap in keywords but does not capture deeper semantic meaning, depth of explanation, or conceptual understanding. Although we benchmarked against randomized text to confirm nonrandom alignment, future studies could use more sophisticated natural language processing techniques (eg, word2vec, BERT) to account for contextual meaning; however, these approaches are not as widely validated for this type of analysis [20-22]. In addition, future studies could incorporate qualitative analyses, such as expert ratings, the DISCERN criteria, and *Journal of the American Medical Association* benchmarks to further delineate the utility of LLMs such as ChatGPT in preclinical medical education.

Second, this study was limited to GPT-4o mini, the free version most accessible to students. Performance may differ with more advanced subscription models. Third, stochasticity in ChatGPT responses means that some outputs contained tangential or extraneous material, which may have modestly influenced similarity scores.

Additionally, this study examined only 5 preclinical subject areas; inclusion of additional subject areas such as anatomy or physiology could further clarify ChatGPT’s role across the broader preclinical curriculum. Finally, the rapid evolution of LLMs means that ChatGPT’s performance may change over time. As newer versions are released, response accuracy, style, and alignment with reference sources like UpToDate may differ, potentially limiting the reproducibility of our findings in future evaluations.

### Conclusion

In summary, ChatGPT demonstrated meaningful but incomplete similarity to UpToDate across a wide range of preclinical topics, with the strongest performance in pharmacology. These findings suggest that ChatGPT can serve as a useful adjunctive tool for medical students during the preclinical phase, provided its limitations are recognized and its use is coupled with authoritative references. As generative AI continues to evolve, collaboration between educators, clinicians, and developers will be essential to harness its potential while safeguarding accuracy, transparency, and educational value.

## Supplementary material

10.2196/82885 Multimedia Appendix 1 Supplementary text detailing the methodology used in the study.

10.2196/82885 Multimedia Appendix 2 Precise Values for Figures 2-6

## Abbreviations

AI: artificial intelligence
LLM: large language model
TF-IDF: term frequency–inverse document frequency
