# Supplementary material for: ChatGPT versus UpToDate in Preclinical Medical Education: Cross-Sectional Analysis Using Term Frequency–Inverse Document Frequency Cosine Similarity
Source: JMIR Med Educ. 2026 Mar 20;12:e82885. doi: 10.2196/82885 (PMC13004592; doi:10.2196/82885)
Supplement: Multimedia Appendix 1 [file mededu-v12-e82885-s001.docx]

# Methodology Overview

This comprehensive methodology involves three critical steps:

1. Response Normalization
2. Random Samples
3. Cosine Similarity Scoring

## Supplemental Content 1: Response Normalization

Utilizing the [spaCy en_core_web_sm](https://spacy.io/models/en) natural language processing model, we standardize each response as follows:

- **Tokenization**: Each response, regardless of source, is broken down into individual tokens.
- **Stop Words and Punctuation Removal**: Common stop words, punctuation, numerical values, and extra whitespace are removed from the tokens.
- **Lemmatization**: Each token is reduced to its lemma, or base form, ensuring consistency across terms.

## Supplemental Content 2: Random Sample Generation

1. **Corpus Construction**: Let $C=\{d*1,d_{2},\ldots,d_{N}\}$ be the corpus composed of $N$ documents, each normalized to a set of terms (as described in _Supplemental Content 1*).
2. **Term Distribution**: Let $U$ be the number of unique terms in $C$. The probability $P\left( t \right)$ of selecting any term $t$ from $C$ is:

$$P\left( t \right)=\frac{1}{U},\text{for}t\in\text{unique terms of}C.$$

3. **Length Distribution**: Let $L=\{l_{1},l_{2},\ldots,l_{N}\}$ be the list of lengths where $l_{i}=\text{length}\left( d_{i} \right)$ represents the length of each document $d_{i}$.
4. **Generating Random Samples**: To create a random sample $S_{j}$ (with $j\in\{1,2,\ldots,1000\}$):
   - Choose a length $l_{j}$ randomly from $L$ using a uniform distribution.
   - Construct $S_{j}$ by selecting $l_{j}$ terms independently and randomly from the term distribution $P\left( t \right)$.

- The resulting sample $S_{j}$ can be represented as:

$$S_{j}=\{t_{1},t_{2},\ldots,t*l_{j}\},\text{where}t_{k}\sim P\left( t \right),k=1,\ldots,l_{j}.$$

This describes how to generate 1000 random samples, each formed by randomly selecting terms according to the defined term and length distributions.

## Supplemental Content 3: Cosine Similarity Scoring

Using the TfidfVectorizer from [scikit-learn](https://scikit-learn.org/1.5/modules/generated/sklearn.feature_extraction.text.TfidfVectorizer.html), we vectorize the responses for all of the upToDate response, Chat GPT responses and Random samples

From here, we can compute the cosine similarity between different groups of TF-IDF vectors:

- UpToDate vs. Chat GPT
- Random vs. Chat GPT

### Mathematical Representation of Cosine Similarity

Given two vectors $\overset{\to}{u}$ and $\overset{\to}{v}$ representing the TF-IDF vectors of two responses, the cosine similarity $cosine_{s}im\left( \overset{\to}{u},\overset{\to}{v} \right)$ is defined as:

Where:

$\overset{\to}{u}\cdot\overset{\to}{v}$ is the dot product of the vectors.

$\left| \overset{\to}{u} \right|$ and $\left| \overset{\to}{v} \right|$ are the Euclidean norms of $\overset{\to}{u}$ and $\overset{\to}{v}$, respectively.

### Group Similarity Calculations

UpToDate vs. Chat GPT: Compute the cosine similarity for each pair of vectors in the Google and Chat GPT response groups. Flatten the matrix of similarity scores and calculate the average and standard deviation:

Random vs. Chat GPT: Compute the cosine similarity for each pair of vectors between the random samples and Chat GPT responses. Flatten the similarity scores and take the upper 99.999th percentile to establish a statistical significance baseline.

## Per-Question Analysis

All analysis is performed on a per-question basis to ensure granularity and targeted insights.
