# Supplementary material for: ChatGPT versus UpToDate in Preclinical Medical Education: Cross-Sectional Analysis Using Term Frequency–Inverse Document Frequency Cosine Similarity
Source: JMIR Med Educ. 2026 Mar 20;12:e82885. doi: 10.2196/82885 (PMC13004592; doi:10.2196/82885)
Supplement: Multimedia Appendix 2 [file mededu-v12-e82885-s002.docx]

Figure 2 Values

| **Question** | **Average Cosine Similarity** |
| --- | --- |
| **What amino acid is the precursor to histamine?** | 0.02764997938142850 |
| **What amino acids are negatively charged at physiologic pH?** | 0.3878639365782860 |
| **What amino acids are positively charged at physiologic pH?** | 0.36482069695132200 |
| **What is the role of hexokinase?** | 0.10811460733182800 |
| **What is the cause of hereditary fructose intolerance?** | 0.5658522534493670 |
| **What is Vitamin B12 the cofactor for?** | 0.2502297298242360 |
| **What is euchromatin?** | 0.28433521162509600 |
| **what are missense mutations?** | 0.2593250071273030 |
| **What is maple syrup urine disease?** | 0.2755879390150890 |
| **What is the pathophys pyruvate kinase deficiency?** | 0.2783666653800680 |
| **What cancers are seen with BRCA1/2 mutations?** | 0.4053816936153320 |
| **What is the pathophysiology of defective mismatch repair?** | 0.3564996826700570 |
| **What are common inborn errors of metabolism?** | 0.3308376303207620 |
| **What are symptoms of mucopolysaccharidoses?** | 0.1487669038114980 |
| **What is acute intermittent porphyria?** | 0.20774015968238500 |
| **What are the lab findings of sideroblastic anemia?** | 0.4369307823979960 |
| **Whatare are intermediates in b12 and folate metabolism?** | 0.1681052178626680 |
| **How does diabetic ketoacidosis occur?** | 0.2030976216117520 |
| **What vitamin deficiencies occur with pancreatic insufficiency?** | 0.38063868663349500 |
| **What mutation is seen with hemochromatosis** | 0.31464690027705700 |
| **What occurs with thiamine deficiencies?** | 0.19125316976087300 |
| **How does aldosterone increase fluid retention?** | 0.1978584424392570 |
| **What is the pathophysiology of SIADH?** | 0.33756730482948400 |
| **What is phosphoenolpyruvate carboxykinase deficiency?** | 0.38287314951783500 |
| **What are some mitochondrial diseases?** | 0.3526215607433520 |
| **How does genetic anticipation work?** | 0.2575795866563430 |
| **How does ethylene glycol poisoning occur?** | 0.4770394567115300 |
| **How does beta thalassemia occur?** | 0.5195762508740300 |
| **What is the pathophysiology of fatty acid oxidation disorders?** | 0.17533777834587700 |
| **what are nonsense mutations?** | 0.23131633785665000 |

Figure 3 Values

| **Question** | **Average Cosine Similarity** |
| --- | --- |
| **How do type 1 hypersensitivity reactions occur?** | 0.2969905373908600 |
| **what are examples of type 4 hypersensitivity reactions?** | 0.20250758671505000 |
| **What occurs during CD4+ t cell activation** | 0.3826605546668060 |
| **What does TGF-B do?** | 0.28347226928598700 |
| **What stimulates eosinophils?** | 0.15832181951307000 |
| **What is the innate immune system?** | 0.481993210406684 |
| **What are toll like receptors?** | 0.3729576328645770 |
| **How do CTLA-4 receptors do negative regulation?** | 0.4208346608790140 |
| **What does IL-10 do?** | 0.36644219671702100 |
| **What are the biomarkers for rheumatoid arthritis?** | 0.29241655441908500 |
| **What are natural killer T cells?** | 0.5066024124839800 |
| **What is B cell exhaustion?** | 0.2865972208266640 |
| **What occurs with IgA deficiency?** | 0.21923029993667300 |
| **What is severe combined immunodeficiency?** | 0.240096774888059 |
| **What are concerning diseases when CD4 < 200?** | 0.24404794653665800 |
| **What are the serologic findings during an acute chronic Hep B infection?** | 0.4170788981431380 |
| **What are neutrophil extracellular traps?** | 0.1490344839889110 |
| **What type of white blood cell is most common normally?** | 0.05461677430226030 |
| **What do basophils do?** | 0.18516215952980000 |
| **What testing shoudl be done to diagnose sjogren?** | 0.12810879133935200 |
| **How does vitamin A deficiency affect the immune system?** | 0.2603402077348090 |
| **How does colchichine work?** | 0.30437381780386300 |
| **What are causes of neutropenia?** | 0.36367281810842200 |
| **What is molecular mimicry?** | 0.1839111994965690 |
| **What is a superantigen?** | 0.22435298033763100 |
| **What is C3 deficiency?** | 0.21093633449604100 |
| **What pathogens does MAC complex deficiency predispose to?** | 0.27723246800884600 |
| **What is B cell class switching?** | 0.3178677172444320 |
| **How do leukocyte adhesion deficiency type 3 present?** | 0.22663389314839800 |
| **What is selective IgM deficiency?** | 0.19526501012930200 |

Figure 4 values

| **Question** | **Average Cosine Similarity** |
| --- | --- |
| **What is primary syphilis?** | 0.3301118566188070 |
| **What is the pathogenesis of tetanus?** | 0.25741991571435000 |
| **What are Shiga toxin producing E coli complications?** | 0.2600790688926390 |
| **What is shiga toxin?** | 0.5159917492297360 |
| **What is moraxella catarrhalis?** | 0.3114553839739180 |
| **What is the malaria life cycle?** | 0.44330064804705200 |
| **What is bacterial transformation?** | 0.5430946978425260 |
| **What is aflatoxin a risk factor for?** | 0.13220093585175300 |
| **What is the treatment for neurocystercosis?** | 0.26301322300934 |
| **What is the treatment for histoplasmosis?** | 0.21767950251265400 |
| **What are symptoms of disseminated gonorrhea?** | 0.267686915806831 |
| **What is giardiasis?** | 0.269236840828465 |
| **What are complications of strep pyogenes pharyngitis?** | 0.31809534645231400 |
| **What are diseases seen when CD4 < 50** | 0.21612545617383200 |
| **What are the symptoms of primary HIV infection** | 0.18376089326167100 |
| **What is the life cycle of strongyloides stereocolis?** | 0.5698082586099030 |
| **What age is clostridium botulism most concerning in?** | 0.4123947085498960 |
| **What are the imaging findings of cerebral toxoplasmosis?** | 0.37085727492983300 |
| **What treatment is recommended for acute simple cystitis in adult and adolescent males?** | 0.28103663147557600 |
| **What are the skin findings of typhoid fever?** | 0.20490053017952900 |
| **What are the symptoms of coccidoidal meningitis?** | 0.21861945940274000 |
| **What are the drugs used to treat pseudomonas?** | 0.1884656072396230 |
| **What is a risk factor for actinomyces infection?** | 0.21680246675753100 |
| **What is kaposi sarcoma?** | 0.2040438080546860 |
| **What are the TORCH infections?** | 0.3497923279845110 |
| **What is Lemierre syndrome?** | 0.3403771638523940 |
| **What are the complications of disseminated tuberculosis?** | 0.20344985653590300 |
| **How does H ducreyi present?** | 0.3636988479278390 |
| **What are the complications of Chagas disease?** | 0.26579582950942200 |
| **What are the symptoms of ehrlichiosis?** | 0.18181203339508800 |

Figure 5 values

| **Question** | **Average Cosine Similarity** |
| --- | --- |
| **What is coagulative necrosis?** | 0.12149576720784200 |
| **What is Virchow's triad?** | 0.3972910845118510 |
| **What is fibrinous necrosis?** | 0.08739665494013890 |
| **What is the APC/beta catenin pathway?** | 0.6481775655013230 |
| **What is autosomal dominant polycystic kidney disease?** | 0.45441351707563700 |
| **What is the translocation for AML?** | 0.3236175457795280 |
| **What are small round blue cell tumors?** | 0.42959011693141200 |
| **What are paraneoplastic syndromes?** | 0.24424735772527700 |
| **What is myelodysplasia?** | 0.29799726677756700 |
| **Where do lacunar infarcts occur?** | 0.19277318669420800 |
| **What is the histology seen in celiacs disease?** | 0.24248372666925000 |
| **What causes achalasia?** | 0.5005666923276510 |
| **What is AA amyloidosis?** | 0.5388695370368250 |
| **What are teratomas?** | 0.22994799105304700 |
| **What is seen with fibrocystic change?** | 0.5590497226123240 |
| **What is the pathophysiology of post-cardiac injury syndromes?** | 0.4325311720965750 |
| **What are the clinical features of Dressler syndrome?** | 0.2570739224063320 |
| **What are the mechanisms of hypercalcemia?** | 0.24932890050972100 |
| **When does hemosiderin accumulate in macrophages?** | 0.2937789882156700 |
| **What finding on blood smear is characteristic of HELLP syndrome?** | 0.1932201650253440 |
| **glanzmann thrombasthenia is a defect in what?** | 0.09875488635979210 |
| **What disese presents with hypersegmented neutrophils?** | 0.30882722340369900 |
| **What are malabsorptive complications of roux-en-y procedures?** | 0.336960929291074 |
| **What are the causes of Bitot spots?** | 0.3373897855287620 |
| **How does IE occur present in IV drug users?** | 0.3571830026084140 |
| **What is the pathophysiology of hydronephrosis and urinary tract obstruction?** | 0.36328451543882800 |
| **What is the clinical prsentation of post-strep glomerulonephritis?** | 0.2632619809615340 |
| **What does tumors secrete alpha fetoprotein?** | 0.17851970681570600 |
| **What is seen with minimal change disease on biopsy?** | 0.5459392333106300 |
| **What is the POTTER sequence?** | 0.16065760264715900 |

Figure 6 values

| **Question** | **Average Cosine Similarity** |
| --- | --- |
| **What is the mechanism of action of diltiazem** | 0.3667259185988920 |
| **What is the treatment for C difficile infection for initial episode?** | 0.29729987394613400 |
| **What is a partial agonist?** | 0.5516011974877080 |
| **What is efficacy?** | 0.15068345193669300 |
| **What is global malabsorption of drugs vs selective?** | 0.40890453311486600 |
| **How do alpha 1 blockers treat bph?** | 0.2653544128231960 |
| **How does insulin cause hypoglycemia?** | 0.42670638628247600 |
| **What are some CYP34A inhibitors?** | 0.138865215954101 |
| **How do Class 1A cardiac drugs work?** | 0.3984313855177600 |
| **What is ion trapping?** | 0.030666379037759100 |
| **What is the treatment for H pylori gastritis?** | 0.33705843860743400 |
| **How does metformin work?** | 0.3047852213457840 |
| **What is the treatment for active tuberculosis?** | 0.4747974992297540 |
| **What drugs cause hypoglycemia?** | 0.45524196626390800 |
| **What is receptor desensitization?** | 0.40900409920005900 |
| **How do ARNIs work?** | 0.5079975247316090 |
| **How do ACEI cause angioedema?** | 0.396508703779024 |
| **When is monteleukast used?** | 0.3863005397448520 |
| **How does coronary steal work?** | 0.3324151274283420 |
| **How is cholinergic toxicity treated?** | 0.3123569136131560 |
| **What are the difference between 1st gen and 2nd gen antihistamine drugs?** | 0.4460760837167210 |
| **What drugs cause delirium?** | 0.06623958332668750 |
| **What are the symptoms of opioid withdrawal?** | 0.297048966511101 |
| **What side effects of opioids are resistant to tolerance?** | 0.2963981556298770 |
| **How is COPD treated?** | 0.21534721832319800 |
| **How do slow release medications work?** | 0.23118506105022400 |
| **How do mRNA vaccines work?** | 0.3727482400541060 |
| **What are the kinetics of phenytoin elimination?** | 0.6086926856029910 |
| **What is dose dependent response?** | 0.218697960784573 |
| **What is phase I metabolism?** | 0.4480291277911890 |
